# Supplementary material for: In poetry, if meter has to help memory, it takes its time
Source: Open Res Eur. 2023 Feb 23;1:59. Originally published 2021 May 28. [Version 2] doi: 10.12688/openreseurope.13663.2 (PMC10445917; doi:10.12688/openreseurope.13663.2)
Supplement: Peer review history

# Peer review: Reviewer response for version 2

**DOI:** 10.21956/openreseurope.15845.r30896

**Published:** 2023-03-20

**Authors:** Johann-Mattis List

**Affiliations:** 1 Department of Linguistic and Cultural Evolution, Max Planck Institute for the Science of Human History, Jena, Germany

I saw that the authors addressed all my modification requests and suggestions in their new version, and I therefore gladly approve the paper. I am aware that other reviewers might have some reservations against certain methodological aspects, but I am not able to judge the internal discussions here, and independently of those comments, I think that the study merits endorsement, since it discusses a very interesting topic and an interesting experiment that was carried out in order to investigate the topic. If there are fundamental flaws in the argumentation, the future will expose them, but this does not mean that this study does not have a scientific value in itself.

If any, are all the source data and materials underlying the results available?

Partly

Is the work original in terms of material and argument?

Yes

Is the work clearly and cogently presented?

Partly

Is the argument persuasive and supported by evidence?

Partly

Does the research article contribute to the cultural, historical, social understanding of the field?

Yes

Does it sufficiently engage with relevant methodologies and secondary literature on the topic?

Yes

Reviewer Expertise:

NA

I confirm that I have read this submission and believe that I have an appropriate level of expertise to confirm that it is of an acceptable scientific standard.

# Peer review: Reviewer response for version 2

**DOI:** 10.21956/openreseurope.15845.r30899

**Published:** 2023-03-08

**Authors:** Stefan Blohm, Stefano Versace

**Affiliations:** 1 Centre for Language Studies, Radboud University, Nijmegen, The Netherlands; 2 Department of Language and Literature, Max Planck Institute for Empirical Aesthetics, Frankfurt am Main, Germany; 3 Leopardi Centre, University of Birmingham, Birmingham, UK

We thank the authors for the revisions and the responses to our comments. We've thoroughly read the revised version as well as the comments and responses from both the reviewers and the authors.

 The reviews of the previous version had requested a number of clarifications, identified a number of methodological concerns and provided sensible suggestions for addressing them. Unfortunately, none of the major issues have been rectified in the revised version of the article. Specifically, the authors still:

- fail to provide most of the requested clarifications in the article
- draw inferences about participants' cognitive processes from the first author's behaviour
- base their conclusions on inappropriate statistical analyses

As a result, it remains unclear what we can learn from the reported study.

If any, are all the source data and materials underlying the results available?

Partly

Is the work original in terms of material and argument?

Yes

Is the work clearly and cogently presented?

Partly

Is the argument persuasive and supported by evidence?

No

Does the research article contribute to the cultural, historical, social understanding of the field?

No

Does it sufficiently engage with relevant methodologies and secondary literature on the topic?

No

Reviewer Expertise:

SB: language/text comprehension, literary linguistics, poetic form(s); SV: metrics, literary linguistics, Italian literature

We confirm that we have read this submission and believe that we have an appropriate level of expertise to state that we do not consider it to be of an acceptable scientific standard, for reasons outlined above.

# Peer review: Reviewer response for version 1

**DOI:** 10.21956/openreseurope.14736.r28462

**Published:** 2022-03-14

**Authors:** Mara Breen

**Affiliations:** 1 Department of Psychology and Education, Mount Holyoke College, South Hadley, MA, USA

The authors’ major question is whether poetic meter serves as a cognitive schema that supports verbal memory. To assess this claim, they manipulated Italian poems by Dante and Ariosto to have a) consistent meter and rhyme, b) consistent meter but missing rhyme, c) conserved rhyme but incorrect accent pattern, or d) the incorrect # of syllables. These variations were assessed for metric plausibility, and this factor was the predictor in the behavioral assessments. Participants were trained with the poems, then tested on their ability to remember final words. The authors report that metric plausibility predicted reaction time, but only in the manipulations of the Ariosto poems. Greater metric plausibility led to more correct memory of the missing target words, but also led to increased reaction time.

 I can’t recommend this paper pass peer review. I have many questions about the method and results, including how the experiment was run, and the choice of statistical analysis models. In addition, I’m not sure that results are interpretable given the variability in the experimental materials across authors, verses, and manipulations.

**Detailed Comments:**

**Introduction**

 I don’t find that the Introduction as written effectively introduces the research question; nor does it effectively review the relevant literature.

 At the end of the introduction, it’s not clear what is being measured and what is being hypothesized. The authors say that they will “aim at measuring the strength of some metric devices.” but it’s not clear which devices they’re specifically talking about, and, moreover, what specific predictions they’re making about the devices. It would be helpful to see explicit predictions about the pattern of results.

**Methods**

 I don’t understand how the manipulation was implemented in the memory experiment. How were conditions assigned to participants? Was it a Latin Square design? Did each participant encounter the same number of verses from each condition? How many ill-formed versions were participants presented with in total? Were there well-formed fillers included? I worry that if participants heard equal numbers of items from each condition that means that 75% of the items they heard were ill-formed, meaning they would not have a reason to expect well-formed items. If well-formed items were rare (25% of the time), then it’s hard to interpret the reaction time differences as being a result of the manipulation and not a result of metacognitive processes on the part of the participants.

**Test phase**

- How are the participants choosing the muted word? Is it free response or do they choose from a set of alternatives?
- How is reaction time measured? That is, when does the clock start? Are any trials thrown away for having reaction times that are too long? Relatedly, I am quite surprised by the variation in reaction times and how generally long they are. Was there any instruction to the participants to answer as quickly as they could? What do the authors think participants are doing in these very long delays of six or more seconds?

I also don’t understand how the authors are accounting for the fact that the targets across verses are different, both in terms of what the actual words are and the way they were produced across conditions. Speaking as someone who studies the role of prosody in cognitive processing, we know a considerable amount about how the acoustic realization of words contributes to how they are remembered. The fact that the production of the target words is not standardized across conditions (i.e., participants heard different acoustic renditions of the targets across conditions), how can the authors be sure that the differences in memory are due to poetic features?

 I don’t believe the analyses are appropriate here. As I see it, the memory experiment has two outcome variables – accuracy and reaction time. First, for accuracy, I expect to see a mixed-effects logistic regression, where the authors are analyzing outcomes on a trial-by-trials basis, modeling the probability of an accurate response based a set of fixed and random effects. Fixed effects include the experimental condition (NPR, NPA, NPS, ONP), the author (Dante or Ariosto), the metric plausibility as assessed in the pre-test, and any other variables that might influence the result (e.g., lexical frequency or entropy). The random effects should be participant and item (verse). The fixed effects should also include any interactions the authors want to test – for example, the interaction between condition and author.

 For reaction time, the authors should be computing a mixed effects linear regression, modeling the reaction time on a trial-by-trials basis with a response based a set of fixed and random effects. Fixed effects include the experimental condition (NPR, NPA, NPS, ONP), the author (Dante or Ariosto) the metric plausibility as assessed in the pre-test, and any other variables that might influence the result (e.g., lexical frequency, entropy, direction boas). The random effects should be participant and item (verse).

**Data analysis** 

 “The outcome of interest is essentially the presence of a significant correlation between the dependent variables measured in the ranking (the plausibility index, see Figure 2) and in the memory experiment (correct responses, and reaction times, see Figure 4), that would indicate a joint dependence on the type of passage manipulation.”

 Note that there can’t be a correlation between a continuous variable (plausibility index) and a binary variable (correct vs. incorrect).

**Entropy in the accent distribution**.

 It’s not clear why this is being measured. There needs to be some explanation in advance. I am expecting this measure to be included in the analysis somehow, perhaps as a fixed effect in the regression.

**Results**

 This section is not well-written. The authors are mixing results with interpretation, but they should be separated. In addition, as stated above, I don’t believe the analyses are appropriately used here. For example, it’s hard to figure out what is being shown in Figure 4. It appears that the authors have averaged reaction times within conditions, then plotted those averages against the metric plausibility scores. But again, I don’t believe this is the most appropriate analysis as the authors should be determining the simultaneous effect of all predictors on the outcomes using a regression fit over all trials.

**Discussion**

 The first four paragraphs belong in the Introduction as these papers are framing the motivation for the current study. There is also a lot missing here. I would expect for the authors to restate the research question, the hypothesis, and the results. And then attempt to interpret the results in the theoretical framework established in the Intro. Ultimately, it’s not clear what these data are adding to our understanding of how metric structure is processed.

If any, are all the source data and materials underlying the results available?

Yes

Is the work original in terms of material and argument?

Yes

Is the work clearly and cogently presented?

No

Is the argument persuasive and supported by evidence?

No

Does the research article contribute to the cultural, historical, social understanding of the field?

No

Does it sufficiently engage with relevant methodologies and secondary literature on the topic?

No

Reviewer Expertise:

Psycholinguistics, statistical modeling, metric processing

I confirm that I have read this submission and believe that I have an appropriate level of expertise to state that I do not consider it to be of an acceptable scientific standard, for reasons outlined above.

# Response

**Published:** 2022-12-21

**Authors:** Alessandro Treves

**Affiliations:** SISSA, Italy

***Detailed Comments:***

***Introduction***

*I don’t find that the Introduction as written effectively introduces the research question; nor does it effectively review the relevant literature.*

*At the end of the introduction, it’s not clear what is being measured and what is being hypothesized. The authors say that they will “aim at measuring the strength of some metric devices.” but it’s not clear which devices they’re specifically talking about, and, moreover, what specific predictions they’re making about the devices. It would be helpful to see explicit predictions about the pattern of results.*

**Response: **We thank the reviewer for her observation, however we think that the three components we consider, of what we broadly refer to as meter, are rather emphatically salient throughout the paper. As to hypothesis-testing science, it is definitely not our style, but if we can mention expectations, then clearly our expectations about the role of meter in Dante were not met by our findings. We thank the reviewer for stimulating us to make these disappointed expectations clearer in the revision.

***Methods***

*I don’t understand how the manipulation was implemented in the memory experiment. How were conditions assigned to participants? Was it a Latin Square design? Did each participant encounter the same number of verses from each condition? How many ill-formed versions were participants presented with in total? Were there well-formed fillers included? I worry that if participants heard equal numbers of items from each condition that means that 75% of the items they heard were ill-formed, meaning they would not have a reason to expect well-formed items. If well-formed items were rare (25% of the time), then it’s hard to interpret the reaction time differences as being a result of the manipulation and not a result of metacognitive processes on the part of the participants.*

**Response: **Indeed, the design can be loosely called a Latin Square, although each participant is only tested on a row of the square. The crucial element to make it balanced is that the 24 participants for each author exhaust the 24 possible rows. This is now better explained and the term Latin Square is included to facilitate the reading: For the experiment, every subject was administered four texts in total, by the same (original) author: one per canto passage and one per condition (a Latin square design). Therefore, twenty-four combinations were created. Although 3 of the 4 versions could be construed to be ill-formed on metrical grounds, as the reviewer notes, we did make an effort to make the manipulations soft – as the revised text emphasizes – and the plausibility rankings show that they were indeed perceived as such.

**Test phase**

- *How are the participants choosing the muted word? Is it free response or do they choose from a set of alternatives?*

**Response: **The text says: Three written alternatives appeared on the screen and the participant had to read aloud his/her choice.

- *How is reaction time measured? That is, when does the clock start? Are any trials thrown away for having reaction times that are too long? Relatedly, I am quite surprised by the variation in reaction times and how generally long they are. Was there any instruction to the participants to answer as quickly as they could? What do the authors think participants are doing in these very long delays of six or more seconds?*

**Response: **There were no instructions that they had to answer as quickly as possible. Considering the task, and the processing cost it takes, we are not particularly surprised by differences in time: some participants enjoyed reverberating the verses more than others; what is indicative, given the balanced design, are the differences across conditions. Outliers were already excluded in the analysis as previously reported.  

*I also don’t understand how the authors are accounting for the fact that the targets across verses are different, both in terms of what the actual words are and the way they were produced across conditions. Speaking as someone who studies the role of prosody in cognitive processing, we know a considerable amount about how the acoustic realization of words contributes to how they are remembered. The fact that the production of the target words is not standardized across conditions (i.e., participants heard different acoustic renditions of the targets across conditions), how can the authors be sure that the differences in memory are due to poetic features?*

**Response: **The muted/target words were actually the same across conditions, as already noted: Notably, for every passage we chose options which were consistent across all conditions, allowing a fair comparison in the results. As the reviewer observes, clearly altering the accent pattern, or the number of syllables, or ablating the rhymes will have affected in subtle ways the way the actor recited the non-poems, despite the target words being overtly untouched by the manipulations. This is part of the effects we aimed to measure: we do not distinguish in this study between the effects of the alterations that would be evident in a written transcription and those that only emerge from the acoustically expressed verse prosody.

*I don’t believe the analyses are appropriate here. As I see it, the memory experiment has two outcome variables – accuracy and reaction time. First, for accuracy, I expect to see a mixed-effects logistic regression, where the authors are analyzing outcomes on a trial-by-trials basis, modeling the probability of an accurate response based a set of fixed and random effects. Fixed effects include the experimental condition (NPR, NPA, NPS, ONP), the author (Dante or Ariosto), the metric plausibility as assessed in the pre-test, and any other variables that might influence the result (e.g., lexical frequency or entropy). The random effects should be participant and item (verse). The fixed effects should also include any interactions the authors want to test – for example, the interaction between condition and author.*

**Response: **We ask in the Discussion "Can the hypothesis of differential schema activation be tested experimentally?" as a speculation, and to encourage further work, also by others, but our study is hypothesis-free, which we believe to be one of its strengths. As discussed in the response to the other reviewers, the manipulations were intended to be soft, and indeed the shuffling analysis now reported in the extended data confirms that only the NonPoem without Rhymes sounded significantly abnormal: In terms of significance, we do report the significance and non-significance of the main finding, the plausibility-memory score correlations for Ariosto and Dante, respectively, but we believe readers remain free to interpret the data however they prefer. See linked figure here.

*For reaction time, the authors should be computing a mixed effects linear regression, modeling the reaction time on a trial-by-trials basis with a response based a set of fixed and random effects. Fixed effects include the experimental condition (NPR, NPA, NPS, ONP), the author (Dante or Ariosto) the metric plausibility as assessed in the pre-test, and any other variables that might influence the result (e.g., lexical frequency, entropy, direction boas). The random effects should be participant and item (verse).*

**Response: **We do report the wide distribution of reaction times, which makes the shuffling analysis redundant: again, we do report the significance of the correlation …in each of the four types of trials above, the more metrically plausible the passage, the longer the reaction time. However, the trend is significant only with Ariosto, if data from the two authors are analyzed separately, and it is significant overall (p<0.004) with a slope mainly determined by the Ariosto data, if analyzed together, as shown in Figure 4 (linked here). The slope for the Dante data alone would be higher, but not significant, likely because of the limited plausibility range spanned.

***Data analysis*** 

 “The outcome of interest is essentially the presence of a significant correlation between the dependent variables measured in the ranking (the plausibility index, see Figure 2) and in the memory experiment (correct responses, and reaction times, see Figure 4), that would indicate a joint dependence on the type of passage manipulation.”

*Note that there can’t be a correlation between a continuous variable (plausibility index) and a binary variable (correct vs. incorrect).*

**Response: **In fact, the memory score is not a binary variable, as shown clearly in Fig. 4: it spans a range from 31 to 45.

***Entropy in the accent distribution*** *.*

*It’s not clear why this is being measured. There needs to be some explanation in advance. I am expecting this measure to be included in the analysis somehow, perhaps as a fixed effect in the regression*.  

**Response: **The two entropy measures we present are two possible synthetic descriptions of the variability of accent patterns across the 4 passages we have selected for each author. Other measures are of course possible. We now cite another one from a recent paper: These two entropy measures appear no less sensitive, with our passages, than others recently proposed (see e.g. Sela & Gronas, 2022). but of course any such measure can only give a rough indication. It is also worthwhile to note, particularly to anglophone readers, that accent patterns are much less defined and less binary in Italian, a language with less marked consonant dominance (Ramus et al, 1999)  

 Ramus, F., Nespor, M., & Mehler, J. (1999). Correlates of linguistic rhythm in the speech signal. Cognition, 73(3), 265-292.

***Results***

*This section is not well-written. The authors are mixing results with interpretation, but they should be separated. In addition, as stated above, I don’t believe the analyses are appropriately used here. For example, it’s hard to figure out what is being shown in Figure 4. It appears that the authors have averaged reaction times within conditions, then plotted those averages against the metric plausibility scores. But again, I don’t believe this is the most appropriate analysis as the authors should be determining the simultaneous effect of all predictors on the outcomes using a regression fit over all trials.*  

**Response: **We have taken, as always in our research, a hypothesis-free approach, which leaves readers free to interpret the observations as they wish. If anything, again, our expectations were contradicted by the findings.

***Discussion***

*The first four paragraphs belong in the Introduction as these papers are framing the motivation for the current study. There is also a lot missing here. I would expect for the authors to restate the research question, the hypothesis, and the results. And then attempt to interpret the results in the theoretical framework established in the Intro. Ultimately, it’s not clear what these data are adding to our understanding of how metric structure is processed.*  

**Response: **We feel the previous response applies to this criticism as well. Maybe one indication emerging from these data is precisely that there is no unique way metric structure is processed, as passages with similar meter are differently affected by our delicate manipulations.

# Peer review: Reviewer response for version 1

**DOI:** 10.21956/openreseurope.14736.r28183

**Published:** 2022-01-27

**Authors:** Johann-Mattis List

**Affiliations:** 1 Department of Linguistic and Cultural Evolution, Max Planck Institute for the Science of Human History, Jena, Germany

I read the study *In poetry, if meter has to help memory, it takes time* by Andreetta *et al*. with great interest. Poetry, reflected in meter and rhyme, plays an important role in historical linguistics, my own field of research. The analysis of rhyme patterns allows us to reconstruct ancient stages of a language which might not be reflected in written sources (see Baxter 1992 for an overview on Old Chinese phonology). Assuming that meter often reflects – at least to a certain degree – the prosody of a language may allow us to reconstruct prosodic structures for languages with attested poetic traditions (Kümmel 2018). Given the importance of poetry, meter, and rhyme for a variety of scientific fields, the study conducted by the authors is of great value and has the potential to inspire follow-up studies.

 Due to my background in historical linguistics and computational linguistics, I do not feel confident to comment on questions of the experimental design or the interpretation of the results. As a result, I will try to provide comments which may help the authors to make their study more accessible to a broader audience and improve its transparency.

 First, I would suggest that the authors clarify the process of poem manipulation a bit more. They give one figure as an example, but I find it hard to interpret the figure, since I did not really understand which parts are manipulated and which parts are original. Investing more time in the design of the figure is probably worthwhile, as it would help the readers to understand more clearly what happens with the poems, where pseudowords are inserted, etc. Given that the examples are in Italian (as far as I asssume, even in a historical Italian variety that is far from being used today), I’d furthermore suggest to provide translations (maybe even using English pseudowords) of all passages (including the data showing in the supplementary PDF).

 Second, I’d like to encourage the authors to explain a bit more about the sociolinguistic role which Dante and Ariosto play in Italy today. If I understand this correctly, both text must look rather archaic to modern language users, so I wonder to which degree the application of ancient rather than modern poetry might have had an impact on the results.

 Third, I would like to encourage the authors to share some more information on *Schema theory* and some other terms and frameworks they use. As far as my reading of their text is concerned, I had the impression that the authors assume that their readers will know these terms, so it would not be important to explain them in more detail, but given that their study may be interesting for a broader reading circle, it may be worthwile to rephrase and extend the introduction and also the discussion to be more inclusive with respect to readers from different scientific backgrounds.

 Fourth, I found the data not very well explained, as already mentioned in my comment made on the article earlier:

 What I miss from the current study, however, are more explicit explanations on the data which you have shared (detailed description of column names, which information is used where in the article, etc.), and also that you share more detailed information on the software that was used for plotting. For example, you mention the use of the SUBTLEX-IT data for assessing word frequencies, but I had to look quite a bit when I was trying to find where in the data files you had this information provided. In order to avoid that readers interested in the details of your methods have to second-guess what part of the data relates to what part of the article, it is always recommended to be very verbose about the data, ideally providing a README file that provides all necessary information, specifically explaining what one can find in which column. As a scientist who has been struggling a lot with studies in which code is not being shared fully, I’d also recommend to share your plotting code for the individual data plots, also in order to allow young scholars to learn from your expertise.

 To summarize my comment here: the supplementary data should be *explained* more transparently, ideally by double-checking with the FAIR principles of data sharing (Wilkinson *et al*. 2016). Additionally, I’d ask the authors to also share the code they used for their plots, as this is a major requirement for replicability and it also helps younger scholars not experienced in doing plots and the like, to learn from the authors in their own work.

 As a fifth and last point, I’d like to ask the authors to which degree they have tried to make sure that the pseudowords they use are neutral across the poems: could it be possible that by coincidence they selected pseudowords that differ with respect to their memorizability, e.g., because they are more or less phonetically isolated? I do not know if studies on this question exist, but I could imagine that certain pseudowords are harder to learn than others, maybe because they are phonotactically less common. If the author know of studies that have looked into such differential characteristics of pseudowords, it may be useful to discuss them quickly.

If any, are all the source data and materials underlying the results available?

Partly

Is the work original in terms of material and argument?

Yes

Is the work clearly and cogently presented?

Partly

Is the argument persuasive and supported by evidence?

Partly

Does the research article contribute to the cultural, historical, social understanding of the field?

Yes

Does it sufficiently engage with relevant methodologies and secondary literature on the topic?

Yes

Reviewer Expertise:

historical linguistics, computational linguistics, digital humanities

I confirm that I have read this submission and believe that I have an appropriate level of expertise to confirm that it is of an acceptable scientific standard, however I have significant reservations, as outlined above.

## References

[1] : A Handbook of Old Chinese Phonology. *de Gruyter.* .1992;

[2] : “Silbenstruktur Und Metrik: Neues Zum Altavestischen” In eds O. Hackstein, D.Gunkel Language and Meter. *Brill* .2018;129-57

[3] : The FAIR Guiding Principles for scientific data management and stewardship. *Sci Data* .2016;3: 10.1038/sdata.2016.18 160018 10.1038/sdata.2016.18 26978244PMC4792175

# Response

**Published:** 2022-12-21

**Authors:** Alessandro Treves

**Affiliations:** SISSA, Italy

*First, I would suggest that the authors clarify the process of poem manipulation a bit more. They give one figure as an example, but I find it hard to interpret the figure, since I did not really understand which parts are manipulated and which parts are original. Investing more time in the design of the figure is probably worthwhile, as it would help the readers to understand more clearly what happens with the poems, where pseudowords are inserted, etc. Given that the examples are in Italian (as far as I asssume, even in a historical Italian variety that is far from being used today), I’d furthermore suggest to provide translations (maybe even using English pseudowords) of all passages (including the data showing in the supplementary PDF).*

**Response:** We thank the reviewer for this suggestion, expressed also by the other reviewers, to which we have responded by emphasizing the attempt to keep manipulations minimal, by better explaining the accent alterations, and by clarifying that muted non-words were chosen to be the same across conditions, and in positions other than where the manipulations occurred. See also the annotated pdf in the revised Extended Data. We refrain from providing translations in English of the surviving words, because they would look awkward interspersed with the non-words, but the translations of the original Dante and Ariosto passages are of course readily available online, in a number of different variants – how to translate them has been a major issue in itself.

*Second, I’d like to encourage the authors to explain a bit more about the sociolinguistic role which Dante and Ariosto play in Italy today. If I understand this correctly, both text must look rather archaic to modern language users, so I wonder to which degree the application of ancient rather than modern poetry might have had an impact on the results.*

**Response: **Indeed, this is an important point, which it is difficult to discuss in a few sentences, given also the heterogeneity of the young Italian population and of their educational experiences. Broadly speaking, Dante remains, even among many who have really not studied his poetry in school, the respected avuncular figure of a genius who, almost single-handedly, with his creativity made the Florentine dialect into standard Italian. The celebration of 700 years from his death stimulated several festive initiatives around the country, with public readings, etc. Ariosto is nowadays much less read, but easier to read and quite enjoyable. They can both be argued to be associated, in the public imagination, with notions of boundless imagination and freedom, in contrast to the often oppressive normativity and rule-learning associated e.g. to Latin grammar, in the traditional educational framework. We enjoy the opportunity to exchange this comments with the reviewer, but feel that they would be somewhat out of place, without proper evidence to support them, in a scientific paper.  

*Third, I would like to encourage the authors to share some more information on Schema theory and some other terms and frameworks they use. As far as my reading of their text is concerned, I had the impression that the authors assume that their readers will know these terms, so it would not be important to explain them in more detail, but given that their study may be interesting for a broader reading circle, it may be worthwile to rephrase and extend the introduction and also the discussion to be more inclusive with respect to readers from different scientific backgrounds.*

**Response: **Thank you for this other important comment, which have addressed by substantially revising the two relevant paragraphs: A schema, whether directly functional like those involved in preparing coffee (Norman & Shallice, 1986) or social/ornamental, like rituals of salutations (Taylor & Crocker, 1981), can be considered as a set of regularities that help organize and retrieve information (Van Kesteren et al., 2012). Its neural basis would be the memory trace of those regularities, that helps funnel neural activity so as to reinstate them. In language processing, “narrative schemata” have often been described, as the features of stories which make them easier to remember, sometimes called “story grammars” (Rumelhart, 1975). In the present context, however we are considering metrical schemata: patterns which, by somewhat restricting options and encouraging expectations, facilitate the recall of verses. Thus, meter (broadly defined) should help us recruit, and possibly produce, the next element of a sequence stored in our memory. In facilitating verbal sequence replay, metrical features appear to be effective with extended “trajectories”, lasting even several verses. These are extended relative to the short trajectories thought to be produced by the phonological loop of Baddeley’s model of working memory, which are presumed to last only a couple of seconds, precisely because of the lack of specific devices that extend their range (Baddeley, 1992; Haluts et al, 2020).

*Fourth, I found the data not very well explained, as already mentioned in my comment made on the article earlier:*

*What I miss from the current study, however, are more explicit explanations on the data which you have shared (detailed description of column names, which information is used where in the article, etc.), and also that you share more detailed information on the software that was used for plotting . For example, you mention the use of the SUBTLEX-IT data for assessing word frequencies, but I had to look quite a bit when I was trying to find where in the data files you had this information provided.* *In order to avoid that readers interested in the details of your methods have to second-guess what part of the data relates to what part of the article, it is always recommended to be very verbose about the data, ideally providing a README file that provides all necessary information, specifically explaining what one can find in which column. As a scientist who has been struggling a lot with studies in which code is not being shared fully, I’d also recommend to share your plotting code for the individual data plots, also in order to allow young scholars to learn from your expertise.*

**Response: **We thank the reviewer for this suggestion, a README file is now included in the supplementary material, where indications about what each column represents are provided. The Extended Data are also now clearer.

*To summarize my comment here: the supplementary data should be explained more transparently, ideally by double-checking with the FAIR principles of data sharing (Wilkinson et al. 2016). Additionally, I’d ask the authors to also share the code they used for their plots, as this is a major requirement for replicability and it also helps younger scholars not experienced in doing plots and the like, to learn from the authors in their own work.*

**Response: **Disappointingly perhaps, our plots were simply made in Excel, and assembled in Powerpoint, also for ease of communication among us. We thank the reviewer for this comment, that made us discover a bug in the published Figure 2, perhaps due to our using pedestrian software: the chance level has dropped below its correct 0.25 value! We do not know at what stage in the article production this happened, and apologize. I will be corrected in the revised version (the figure is correct in our folders).

*As a fifth and last point, I’d like to ask the authors to which degree they have tried to make sure that the pseudowords they use are neutral across the poems: could it be possible that by coincidence they selected pseudowords that differ with respect to their memorizability, e.g., because they are more or less phonetically isolated? I do not know if studies on this question exist, but I could imagine that certain pseudowords are harder to learn than others, maybe because they are phonotactically less common. If the author know of studies that have looked into such differential characteristics of pseudowords, it may be useful to discuss them quickly.*

**Response:** We gratefully acknowledge this comment by the reviewer: this is indeed an aspect we are aware of, but know of no systematic way to handle it, other than relying on our own best judgement. On the one hand, we did our best to manipulate the passages, while on the other trying to avoid an effect of a totally unrelated schema, as we report in the text: an effort was made to minimize the impact of this manipulation (like for the subsequent ones below) by changing phonemes with similar ones, while maintaining the original prosody. Function words were not modified. The degree to which we succeeded can be assessed by readers by looking at the muted words and at their alternatives in the Underlying Data Excel files. It would indeed be nice to be able to rely on more on more objective criteria, but they seem to be a long way behind. For example, arguably the most advanced automated system for generating literary language, GPT-3, has churned out a pathetic attempt when challenged with the incipit of a mere sonnet by Dante (Floridi and Chiriatti, 2020). Clearly, more research is needed in this respect. Floridi, L., & Chiriatti, M. (2020). GPT-3: Its nature, scope, limits, and consequences. Minds and Machines, 30(4), 681-694.

# Peer review: Reviewer response for version 1

**DOI:** 10.21956/openreseurope.14736.r27840

**Published:** 2021-11-15

**Authors:** Stefan Blohm, Stefano Versace

**Affiliations:** 1 Centre for Language Studies, Radboud University, Nijmegen, The Netherlands; 2 Department of Language and Literature, Max Planck Institute for Empirical Aesthetics, Frankfurt am Main, Germany; 3 Leopardi Centre, University of Birmingham, Birmingham, UK

**SUMMARY**

 Focusing on two poetical works of classical Italian literature, the study reported in this article tests the idea that poetic meter and rhyme aid verbal memory.

**Stimulus materials**: The authors selected text sections of 8-9 verse lines from Dante’s *Divina Commedia* and from Ariosto’s *Orlando Furioso*. By substituting individual speech sounds in a portion of lexical words, they converted the original text sections into meaningless (but grammatical) jabberwocky versions that preserved the prosodic structure and the rhyme scheme. These were then modified to yield versions with (a) an irregular number of syllables in some verse lines, (b) an irregular distribution of prominent/accented syllables within some verse lines, and (c) no rhyme in any of the verse lines.

**Procedure/method**: Using audio recordings of the four resulting versions of each text section, the authors conducted two experiments. In one experiment, participants listened to four critical sections of one work (Ariosto: *n*=62; Dante: *n*=65)––each in one of the four experimental conditions––and ranked them according to “poetic plausibility”; resulting rank data were used to calculate the non-linearly weighted average rank per condition: the “metric plausibility index”.

 The main experiment comprised a study phase and a test phase after 24 hours. During study, participants ( *n*=48) listened to four critical sections of one work, each in one of the four experimental conditions. During test, participants were presented with individual lines from the text sections they had heard a day before. In each critical line a single (pseudo-)word was muted, and participants had to choose the muted target word from three alternatives presented on a screen.

**Data analysis**: The authors calculated correlations between the metric plausibility index per condition and the condition means of both the response latencies and the accuracy rates observed in the memory experiment; correlation analyses were conducted separately for the *Divina Commedia* and for *Orlando Furioso*.

**Results**: The authors observed that rankings were sensitive to metrical modifications and particularly to the removal of rhyme. The metrical plausibility index of the *Divina Commedia* was unrelated to either reaction times or response accuracy, whereas the metrical plausibility index of *Orlando Furioso* correlated positively with response accuracy and negatively with reaction times.

**Conclusion**: From these results, the authors concluded that metre facilitates memory retrieval, but that this facilitation requires additional time and cognitive effort.

**COMMENTS**

**Is the work original in terms of material and argument?**

*YES*, because – contrary to prior investigations of accentual-syllabic metre (e.g., Menninghaus et al., 2014; van Peer, 1990) – this study aims to dissociate the metrical constraints on (a) the number of syllables and (b) the distribution of syllable prominence/accent within the verse line.

**Does it sufficiently engage with relevant methodologies and secondary literature on the topic?**

*NO*, because...

 1.  The current version of the article disregards most of the relevant empirical research into memory effects of rhyme and metre (e.g., metre and memory: van Peer, 1990; rhyme and memory: Bower & Bolton, 1969; Lea et al., 2021; Rubin & Wallace, 1989).

 > This concern can be addressed by relating the present study to a broader range of relevant findings (for a recent overview of parallelism-induced memory effects, see Blohm *et al*., 2021).

 2. The authors applied the conversion into pseudo-word verse inconsistently and less systematically than prior investigations relying on this text-modification method (for poetry, e.g., Obermeier *et al*., 2013). Due to these shortcomings it remains unclear in how far the text conversion successfully removed the meaning of the texts; the reported effect of lexical frequency (p. 8 and Fig. 6) casts further doubt on the effectiveness of this procedure. Specifically, modifications:

- Failed to convert all target words of the memory experiment (2/12 in Ariosto and 1/12 in Dante remain unchanged).
- Targeted only a subset of lexical classes, e.g., nouns but not adverbs.
- Targeted only a subset of lexical words per word class.
- Did not substitute all consonants, which is the common procedure.
- Did not substitute speech sounds systematically, e.g., voiceless plosives with voiceless plosives.
- Sometimes resulted in actual words rather than pseudo-words, e.g., *lascio* [I leave] > *bascio* [I kiss]; *lena* [haste] > *rena* [sand].
- Sometimes substituted only a single speech sound per verse line, e.g., *a privilegi **l**enduti e mendaci.*

> Unfortunately, we don't see how this concern could be addressed a posteriori.

**Is the work clearly and cogently presented?**

*PARTLY*, because...

 1. The assumed underlying mechanisms are not made explicit enough.

- The authors appear to assume that activation/recognition of metrical schemata is crucial for facilitated memory retrieval, but how exactly schema recognition is supposed to facilitate the retrieval of pseudo-words remains opaque. Clearly, the pseudo-word targets cannot be part of the metrical schema.
- On page 10, the authors further point to Rubin's idea that the constraints of rhyme restrict the set of alternatives, or possible continuations, in an unfolding sentence/verse (e.g., Bower & Bolton, 1969; Rubin & Wallace, 1989). This explanation seems less plausible for non-lexical items (=pseudo-words) and for the relatively weak constraints of metre, which are consistent with a much larger portion of the lexicon than the constraints of rhyme.

2. The procedure and the analysis are not described in sufficient clarity and detail.

- For instance, it is unclear how participants indicated their ranking in the ranking experiment.
- The authors state (p. 6) that participants navigated through the texts in a self-paced manner but that it was the experimenter who logged participants' responses. Thus it remains unclear how the experiment was controlled, e.g., which machine actually ran the experiment (the experimenter's? the participant's?).
- It is unclear whether the Zoom screen, i.e., the experimenter, was visible at all times during the experiment, and in how far the experimenter interacted with the participants during study and test.

3. Not all decisions regarding the research design and the analysis procedure are sufficiently well motivated. In particular, the current version of the article does not make clear enough...

- Why the authors chose to present jabberwocky verse rather than the original texts?
- Why the authors chose the detour via the metrical plausibility index rather than comparing conditions directly?
- Why the rank data have been transformed? While the authors state (p. 5) that this was intended to accommodate individual participants' uncertainty regarding the ranking, we suspect that this non-linear transformation was applied to make the data fit a priori assumptions: without this non-linear transformation, original pseudo-word versions of *Orlando Furioso* and versions with an irregular number of syllables per line were indistinguishable in terms of metrical plausibility.
- Why the authors opted for an indirect response collection?
- Why the authors chose to vary the number of repetitions per text section during study?
- Why the authors chose to conduct correlation analyses rather than, say, linear and logistic regression analyses?
- Why the authors chose to analyse data from the *Divina Commedia* and from *Orlando Furioso* separately?
- Why the authors report analyses of frequency effects and response bias?

4. The statistical analysis is not described in sufficient detail to allow for replication (e.g., which software was used), and the statistical results are not reported according to the conventional standards of the field (e.g. in the format recommended by the APA). Moreover, the reported results seem to be at odds with the values supplied in the data sheets.

 > These concerns can be addressed by providing more information about (1) the assumed underlying cognitive mechanisms, (2) the experimental procedure, (3) the motivation behind the authors' decisions, and (4) the statistical analysis.

**Is the argument persuasive and supported by evidence?**

*NO*, because of a number of methodological flaws that seriously undermine the validity of the results and of the inferences drawn from them.

 1. The most serious issue relates to the indirect response collection employed in this study. Participants reported their choices to the experimenter who then logged responses via button press. This procedure conflates the behaviour (i.e., latencies and accuracy of responses) of both participants and experimenter. Since it is impossible to dissociate the contributions of participants and experimenter, this conflation renders the behavioural results of the memory experiment uninterpretable.

 > Unfortunately, we don't see how this issue could be successfully addressed.

 2. The sample sizes ( *n*=4) are too small for correlation analyses. This increases the likelihood of both type I and type II errors and inflates the correlation coefficient of significant effects (Aggarwal & Ranganathan, 2016; Knudson & Lindsey, 2014; Makin & Orban de Xivry, 2019).

 > This concern can partly be addressed by pooling all data and by calculating correlations per text section. However, we strongly recommend to conduct ANOVA and/or regression analyses instead in order to test for differences between experimental conditions.

 3. The between-participant design confounds participant group and author, i.e., observed differences between Dante's and Ariosto's verse could in fact merely reflect differences between participants. While the authors maintain that participant groups were indistinguishable in terms of age and sex, they do not provide evidence that groups were indistinguishable in terms of their prior experience with poetry (as assessed e.g. by self report). Prior experience with poetry is necessary for the emergence of the metrical schemata whose activation is assumed to be crucial for facilitated memory retrieval.

 > This concern can be addressed by pooling all data and by not interpreting differences between authors.

**If any, are all the source data and materials underlying the results available?**

*PARTLY*, because the authors do provide the materials and the underlying data. However, it is unclear at times how these data relate to the results reported in the article. For instance, the correlation coefficients reported on page 8 do not match the values supplied in the data sheet.

 > This concern can be addressed by (additionally) providing data in a more user-friendly format, e.g., in a single comma/tab-delimited text file. The reader comment by Johann-Mattis List generally seems to provide sensible suggestions; please also add participant IDs and the number of repetitions during study to the data table.

**Does the research article contribute to the cultural, historical, social understanding of the field?**

*NO*, because – due to the methodological issues outlined above – it remains unclear what we can learn from this study. Unfortunately, the most serious flaw is an inherent feature of the research design and cannot be remedied. We had been enthusiastic to read about this research, but after several thorough readings, we are convinced that the reported results do not support the conclusion that metre takes its time if it has to help memory.

 MINOR POINTS

- End rhyme is not commonly considered a component of metre (cf. Lev Blumenfeld's review report) but rather a para-metrical phenomenon or: a structuring part of the orchestration of the metrical line.
- Extended data: Please underline target words in the supplementary file; please place images next to the text excerpts they were presented with.
- Please make sure to use terminology consistently, e.g., canti/poems/texts.

If any, are all the source data and materials underlying the results available?

Partly

Is the work original in terms of material and argument?

Yes

Is the work clearly and cogently presented?

Partly

Is the argument persuasive and supported by evidence?

No

Does the research article contribute to the cultural, historical, social understanding of the field?

No

Does it sufficiently engage with relevant methodologies and secondary literature on the topic?

No

Reviewer Expertise:

SB: language/text comprehension, literary linguistics, poetic form(s); SV: metrics, literary linguistics, Italian literature

We confirm that we have read this submission and believe that we have an appropriate level of expertise to state that we do not consider it to be of an acceptable scientific standard, for reasons outlined above.

## References

[1] : Sounds funny? Humor effects of phonological and prosodic figures of speech. *Psychology of Aesthetics, Creativity, and the Arts* .2014;8(1) : 10.1037/a0035309 71-76 10.1037/a0035309

[2] : The measurement of metre. *Poetics* .1990;19(3) : 10.1016/0304-422X(90)90023-X 259-275 10.1016/0304-422X(90)90023-X

[3] : Why are rhymes easy to learn?. *Journal of Experimental Psychology* .1969;82(3) : 10.1037/h0028365 453-461 10.1037/h0028365

[4] : Rhyme as resonance in poetry comprehension: An expert-novice study. *Mem Cognit* .49(7) : 10.3758/s13421-021-01167-0 1285-1299 10.3758/s13421-021-01167-0 33893605

[5] : Rhyme and reason: Analyses of dual retrieval cues. *Journal of Experimental Psychology: Learning, Memory, and Cognition* .1989;15(4) : 10.1037/0278-7393.15.4.698 698-709 10.1037/0278-7393.15.4.698

[6] : Sound Shape and Sound Effects of Literary Texts.2021; 10.1515/9783110645958-002 7-38 10.1515/9783110645958-002

[7] : Aesthetic and emotional effects of meter and rhyme in poetry. *Front Psychol* .2013;4: 10.3389/fpsyg.2013.00010 10 10.3389/fpsyg.2013.00010 23386837PMC3560350

[8] : Common pitfalls in statistical analysis: The use of correlation techniques. *Perspect Clin Res* .7(4) : 10.4103/2229-3485.192046 187-190 10.4103/2229-3485.192046 27843795PMC5079093

[9] : Type I and Type II Errors in Correlations of Various Sample Sizes. *Comprehensive Psychology* .2014;3: 10.2466/03.CP.3.1 10.2466/03.CP.3.1

[10] : Ten common statistical mistakes to watch out for when writing or reviewing a manuscript. *eLife* .2019;8: 10.7554/eLife.48175 10.7554/eLife.48175 PMC678526531596231

# Response

**Published:** 2021-12-05

**Authors:** Alessandro Treves

**Affiliations:** SISSA, Italy

We thank the two reviewers for their most attentive reading of our manuscript and their useful comments. Many of the issues they raise cannot, as they note, be addressed ***a posteriori***. Still, we would like to better explain some of the main ***a priori*** choices in our study.

 First, although it is of course impossible to neatly separate semantics from syntactic and metric structure, we tried our best to use our subjective judgement to create original non-poems that strike a balance between removing meaning as much as possible while retaining structure. This motivated sometimes altering voicing and other phonemic features, masterfully used especially by Dante to convey the sense/tone in some verse. The result is in our view an acceptable compromise.

 Second, the choice to conduct the experiment over Zoom sessions was also an acceptable compromise, between the need to reach an adequate number of subjects, in particular during the pandemic, and that of establishing a relationship of trust and common purpose with the experimenter while she (the first author) maintained an objective but caring posture. This is particularly delicate in that we obviously required subjects to have had the exposure to Dante and Ariosto normally available in the Italian school system, without being specialists or, the opposite, harbouring long-lasting negative emotions from such exposure. With our recruitment and Zoom sessions we found that no subject had to be excluded on such basis.

 Third, the training procedure, with a variable number of repetitions depending on the position of each verse in the passage, was designed to facilitate encoding, vaguely mimicking a common rote learning used in school. The proof of its validity was in the pudding, in that subjects achieved memory performances in the intended intermediate range, away from floor and ceiling effects, for the entire length of the passages.

 Regarding the metric plausibility index (which allows an across-subjects correlation with memory performance, whereas comparisons between conditions were within-subjects) it was developed when the study was still limited to Dante, and then applied also to the Ariosto passages.

 Finally, we would like to thank the reviewers for their bibliographic suggestions, some of which we were not aware of, including the recent Blohm *et al* (2021) study, which will undoubtedly be taken into account in our future endeavours.

# Response

**Published:** 2021-12-09

**Authors:** Stefan Blohm

**Affiliations:** Radboud University Nijmegen, The Netherlands

Thanks to the authors for their replies to our review. Unfortunately, the major issues have not been addressed in this response (experimenter-mediated response collection, statistical analysis), but we are confident that this will be rectified in the revised manuscript.

 Considering that the two reviews so far have come to diverging assessments, it might be best to seek a third opinion. Since our criticism focused almost exclusively on the methodology, we trust that the authors will seek a third opinion from a scholar with a solid background in empirical research (e.g., an expert in language-related memory research) to ensure that the scientific quality of this contribution will be properly assessed.

 In the meantime, we provide comments on the points raised in the author response:

1. The authors state that “it is of course impossible to neatly separate semantics from syntactic and metric structure”. The manipulation in question aimed to remove semantics while keeping syntactic and metric structure intact. As prior studies show, it is perfectly possible to do this to the extent that meaning is conveyed by content words, and to do it systematically and exhaustively. Contrary to what is suggested in the reply, there is no need to strike a balance between removing meaning and retaining structure; it is possible to have both if the appropriate procedure is employed. Moreover, the conventional procedure of systematically substituting speech sounds within phoneme classes even allows to keep some sub-phonemic features (e.g., voicing) constant and, in fact, preserves virtually the entire sonority profile of linguistic stimuli.
2. We readily accept that the pandemic situation requires alternative ways of doing experimental research, but we doubt that it necessitates the kinds of choices made in this study (see the comments below). Most importantly: COVID-19 is no excuse for flawed experiment design or inappropriate statistical analysis.
  • It is customary in experimental research that the experimenter refrains from influencing the outcome of the experiment, e.g., by interacting with participants during task performance, or by influencing dependent variables directly. Please note that we do not wish to accuse the first author of consciously manipulating the outcome of the study; however, we feel obliged to point out that it is highly problematic to draw valid conclusions if the experimenter, who is familiar with the hypotheses, modulates the dependent variables
  • As the reported online study demonstrates, it is possible to recruit an adequate number of participants without video conference.
  • Of course, participants should trust that they will be informed about what is expected from them, that the experimenter acts according to accepted scientific criteria, and that the collected data will be handled in compliance with legal and ethical standards. However, we fail to see the need to establish “a relationship of trust and common purpose with the experimenter” beyond that. On the contrary, this is usually seen as an undue influence on participants’ task performance.
  • It is possible to ensure without personal interaction that participants “have had the exposure to Dante and Ariosto normally available in the Italian school system, without being specialists or, the opposite, harbouring long-lasting negative emotions from such exposure”; collecting appropriate self-report measures would have been a straightforward objective alternative.
3. Repeated exposure helps to consolidate memory, and we assume that performance during test varies as a function of the number of repetitions during study. We recommend that the authors make sure to detail in the revised manuscript how a variable vs. fixed number of repetitions facilitates encoding.
4. The authors disclosed in their response that the study had originally been limited to Dante and that the metric plausibility index was introduced only after the Dante study had been completed.
  Frankly, the authors’ a posteriori decisions 1) to extend the experiment after learning that the original study did not yield significant results, 2) to opt for an inappropriate statistical analysis that is prone to yield significant results in the absence of an actual effect, 3) to take the detour via the metric plausibility index (between-participant correlations instead of more convincing within-participant contrasts), and 4) to non-linearly adjust the calculated index to obtain differences between conditions do not increase readers’ confidence in the scientific rigour of this study. Therefore, we recommend that the authors devote particular care during revision to the motivation of these decisions.
  We further recommend that the authors clarify in the article that the Dante sessions were not followed by a ranking experiment (the current version of the article states that participants “were asked to complete this survey after the end of the second session of the main experiment“), and whether all of the ranking data for Ariosto were obtained from participants recruited via Prolific.
5. We are glad to learn that the authors will take the recommended empirical literature into account, and we hope that these suggestions help to better contextualize the current study. However, the authors should not feel obliged to cite our own work unless they consider it relevant to the purpose of their study.

Thanks again to the authors for taking the time to explain some of their decisions in their response. We look forward to reading the revised version of the manuscript.

# Response

**Published:** 2022-12-21

**Authors:** Alessandro Treves

**Affiliations:** SISSA, Italy

***COMMENTS***

***Is the work original in terms of material and argument?***

*YES, because – contrary to prior investigations of accentual-syllabic metre (e.g., Menninghaus et al., 2014; van Peer, 1990) – this study aims to dissociate the metrical constraints on (a) the number of syllables and (b) the distribution of syllable prominence/accent within the verse line.*

***Does it sufficiently engage with relevant methodologies and secondary literature on the topic?***

*NO, because...*

*1.  The current version of the article disregards most of the relevant empirical research into memory effects of rhyme and metre (e.g., metre and memory: van Peer, 1990; rhyme and memory: Bower & Bolton, 1969; Lea et al., 2021; Rubin & Wallace, 1989).*

*> This concern can be addressed by relating the present study to a broader range of relevant findings (for a recent overview of parallelism-induced memory effects, see Blohm et al., 2021).*

**Response:** We thank the reviewer for this suggestion: at the time of submitting this paper we were not aware of this contribution. We have now included it in the discussion: Still, the wide-ranging contribution of sound ‘shape’ to cognitive processes has been noted, in particular in poetry (Blohm et al. 2021) and it might play a role in our findings, in forms not readily evident.

 We have also added the remark that In line with previous studies, we also acknowledge that a potential factor could also be the individual participants’ expertise with poetry and/or music. Such data were not considered, here, and presumably individual differences average out, but these factors are being investigated in a related study.

 2. *The authors applied the conversion into pseudo-word verse inconsistently and less systematically than prior investigations relying on this text-modification method (for poetry, e.g., Obermeier et al., 2013). Due to these shortcomings it remains unclear in how far the text conversion successfully removed the meaning of the texts; the reported effect of lexical frequency (p. 8 and Fig. 6) casts further doubt on the effectiveness of this procedure. Specifically, modifications: *

- *Failed to convert all target words of the memory experiment (2/12 in Ariosto and 1/12 in Dante remain unchanged).*
- *Targeted only a subset of lexical classes, e.g., nouns but not adverbs.*
- *Targeted only a subset of lexical words per word class.*
- *Did not substitute all consonants, which is the common procedure.*
- *Did not substitute speech sounds systematically, e.g., voiceless plosives with voiceless plosives.*
- Sometimes resulted in actual words rather than pseudo-words, e.g., *lascio* [I leave] > *bascio* [I kiss]; *lena* [haste] > *rena* [sand].
- Sometimes substituted only a single speech sound per verse line, e.g., *a privilegi **l**enduti e mendaci.*

**Response:** Each of the points above is a valid concern, which is unfortunately difficult to address *a posteriori*. Satisfying all the listed constraints together, however, would have produced in our judgement repellent non-poems, inappropriate or a study intended to engage, however marginally, the aesthetic enjoyment of subjects drawn from the general Italian population. A useful guide, in this respect, is the success enjoyed by the famous non-poem Il Lonfo, by Fosco Maraini (in F. Maraini, *Gnosi delle fànfole*, Dalai Editore, 1994), in which contagiously enjoyable meta-semantics is achieved by a judicious admixture of words and non-words.

***Is the work clearly and cogently presented?***

*PARTLY, because...*

*1. The assumed underlying mechanisms are not made explicit enough. *

- *The authors appear to assume that activation/recognition of metrical schemata is crucial for facilitated memory retrieval, but how exactly schema recognition is supposed to facilitate the retrieval of pseudo-words remains opaque. Clearly, the pseudo-word targets cannot be part of the metrical schema*.

**Response:** Our report does not discuss, for they would be out of place, models and conjectures about the neural mechanisms involved in the representation of meter. Some of the neural computation research in our group touches on those issues. One general idea, however, is that schemata in general are expressed as rather loose dynamical attractors, which guide the evolution in time of distinct patterns of neural activity to a partial degree, compete with each other, often fail together, and are therefore quite different structure from the rules, with a well-defined domain of application, typically assumed in linguistic analyses. An interesting discussion to be continued elsewhere.

- *On page 10, the authors further point to Rubin's idea that the constraints of rhyme restrict the set of alternatives, or possible continuations, in an unfolding sentence/verse (e.g., Bower & Bolton, 1969; Rubin & Wallace, 1989). This explanation seems less plausible for non-lexical items (=pseudo-words) and for the relatively weak constraints of metre, which are consistent with a much larger portion of the lexicon than the constraints of rhyme*.

**Response:** ​​​​​​​Agreed. The citation of Rubin is not meant to affirm the absolute validity of that explanation in our case, but simply to introduce one relevant and inspiring idea.

 2. *The procedure and the analysis are not described in sufficient clarity and detail. *  

- *For instance, it is unclear how participants indicated their ranking in the ranking experiment*.

**Response:** It is actually written that "At the end they were asked to rank the four poems: from the one they perceived as the best, to the one that sounded worse to them, by simply typing in the platform their preferential order.”

- *The authors state (p. 6) that participants navigated through the texts in a self-paced manner but that it was the experimenter who logged participants' responses. Thus it remains unclear how the experiment was controlled, e.g., which machine actually ran the experiment (the experimenter's? the participant's?).*

**Response:** ​​​​​​​It is actually written that "When tested with the muted non-word (see below), participants would read aloud the left, central or right alternative appearing on the screen, and the experimenter would press the corresponding key (leftward, downward or rightward) on her own keyboard."

- *It is unclear whether the Zoom screen, i.e., the experimenter, was visible at all times during the experiment, and in how far the experimenter interacted with the participants during study and test*.

**Response:** We believe that any doubt about the experimental procedure has been clarified in the revised text.

 3. *Not all decisions regarding the research design and the analysis procedure are sufficiently well motivated. In particular, the current version of the article does not make clear enough... *

- *Why the authors chose to present jabberwocky verse rather than the original texts*?

**Response:** ​​​​​​​This was in fact a rather crucial element of the study: to eliminate or strongly suppress the semantic component, which is so prominent when listening to poetry by both authors. We note most content words were converted into non-words in order to eliminate discernible semantic content, hence semantic effects on memory. We were, in fact, interested in the strength of meter devices, and a semantic effect would have been a major distorting bias.

- *Why the authors chose the detour via the metrical plausibility index rather than comparing conditions directly?*
- *Why the rank data have been transformed? While the authors state (p. 5) that this was intended to accommodate individual participants' uncertainty regarding the ranking, we suspect that this non-linear transformation was applied to make the data fit a priori assumptions: without this non-linear transformation, original pseudo-word versions of Orlando Furioso and versions with an irregular number of syllables per line were indistinguishable in terms of metrical plausibility.*
- *Why the authors opted for an indirect response collection?*
- *Why the authors chose to vary the number of repetitions per text section during study?*
- *Why the authors chose to conduct correlation analyses rather than, say, linear and logistic regression analyses?*
- *Why the authors chose to analyse data from the Divina Commedia and from Orlando Furioso separately?*
- *Why the authors report analyses of frequency effects and response bias?*

**Response:** Overall it feels like the reviewers are asking: why did you prepare couscous with mullet, almonds and saffron rather than spaghetti with anchovies, toasted bread crumbs, capers and olives. They will surely agree that both are nutritious and tasty food to those that like them.

 4. *The statistical analysis is not described in sufficient detail to allow for replication (e.g., which software was used), and the statistical results are not reported according to the conventional standards of the field (e.g. in the format recommended by the APA). Moreover, the reported results seem to be at odds with the values supplied in the data sheets.*

*> These concerns can be addressed by providing more information about (1) the assumed underlying cognitive mechanisms, (2) the experimental procedure, (3) the motivation behind the authors' decisions, and (4) the statistical analysis*.

**Response:** The new figure added to the Extended data with the shuffling analyses should clarify that the statistical analysis was actually very simple. As already pointed out but now further emphasized, our manipulations were delicate and succeeded in not altering much the meter and prosody of our non-poems: only the NPR versions of the Ariosto passages was rated as significantly less plausible at the modest *p* < 0.05  significance level. The memory score differences were similarly limited (see the Extended Data for a comparison with their shuffled distribution). The resulting correlation between the two measures (tight correlation in the case of Ariosto, no correlation for Dante) are presented transparently in Figure 4.  

***Is the argument persuasive and supported by evidence?***

*NO, because of a number of methodological flaws that seriously undermine the validity of the results and of the inferences drawn from them.*

*1. The most serious issue relates to the indirect response collection employed in this study. Participants reported their choices to the experimenter who then logged responses via button press. This procedure conflates the behaviour (i.e., latencies and accuracy of responses) of both participants and experimenter. Since it is impossible to dissociate the contributions of participants and experimenter, this conflation renders the behavioural results of the memory experiment uninterpretable.*

*> Unfortunately, we don't see how this issue could be successfully addressed.*

*2. The sample sizes (n=4) are too small for correlation analyses. This increases the likelihood of both type I and type II errors and inflates the correlation coefficient of significant effects (Aggarwal & Ranganathan, 2016; Knudson & Lindsey, 2014; Makin & Orban de Xivry, 2019).*

*> This concern can partly be addressed by pooling all data and by calculating correlations per text section. However, we strongly recommend to conduct ANOVA and/or regression analyses instead in order to test for differences between experimental conditions.*

*3. The between-participant design confounds participant group and author, i.e., observed differences between Dante's and Ariosto's verse could in fact merely reflect differences between participants. While the authors maintain that participant groups were indistinguishable in terms of age and sex, they do not provide evidence that groups were indistinguishable in terms of their prior experience with poetry (as assessed e.g. by self report). Prior experience with poetry is necessary for the emergence of the metrical schemata whose activation is assumed to be crucial for facilitated memory retrieval.*

*> This concern can be addressed by pooling all data and by not interpreting differences between authors*.  

**Response:** We believe the previous responses addressed the concerns expressed here in somewhat dramatic form. The effect of major first-person expertise at versification is actually a very interesting topic, although we believe not relevant to the participants in this study. It is has been taken up in a separate study by others in our group.

***If any, are all the source data and materials underlying the results available?***

*PARTLY, because the authors do provide the materials and the underlying data. However, it is unclear at times how these data relate to the results reported in the article. For instance, the correlation coefficients reported on page 8 do not match the values supplied in the data sheet.*

*> This concern can be addressed by (additionally) providing data in a more user-friendly format, e.g., in a single comma/tab-delimited text file. The reader comment by Johann-Mattis List generally seems to provide sensible suggestions; please also add participant IDs and the number of repetitions during study to the data table*.  

**Response:** This was done. Thank you for the suggestion.

***Does the research article contribute to the cultural, historical, social understanding of the field?***

*NO, because – due to the methodological issues outlined above – it remains unclear what we can learn from this study. Unfortunately, the most serious flaw is an inherent feature of the research design and cannot be remedied. We had been enthusiastic to read about this research, but after several thorough readings, we are convinced that the reported results do not support the conclusion that metre takes its time if it has to help memory*.  

**Response:** ​​​​​​​We regret the reviewers reached this sad persuasion.

*MINOR POINTS *

- *End rhyme is not commonly considered a component of metre (cf. Lev Blumenfeld's review report) but rather a para-metrical phenomenon or: a structuring part of the orchestration of the metrical line.*

**Response:** ​​​​​​​Agreed. We use the term in a broader sense.

- *Extended data: Please underline target words in the supplementary file; please place images next to the text excerpts they were presented with*.

**Response:** ​​​​​​​Thank you for the suggestion. The Extended Data is now presented in a clearer format

- *Please make sure to use terminology consistently, e.g., canti/poems/texts*.

**Response:** Thank you again. We have revised the wording throughout the text. Now “passages” are the consistently excerpts from specific “canti” and once they are manipulated in different types of “non-poems” they become “texts”.

# Peer review: Reviewer response for version 1

**DOI:** 10.21956/openreseurope.14736.r27806

**Published:** 2021-10-28

**Authors:** Lev Blumenfeld

**Affiliations:** 1 Carleton University, Ottawa, ON, Canada

The article attempts to test the contributions of various aspects of metricality to memory. Non-word metrical passages derived from Dante and Ariosto were manipulated in one of three ways to render them less regular: syllable count, accent distribution, and rhyme. The resulting passages were ranked by listeners, and a metric "plausibility" rating was extracted from those rankings. In the second experiment, a different subjects were presented with the same passages, and tested on their memory retention of one of the words in them. The basic results were: (a) metric plausibility positively correlates with memory retention for Ariosto but not for Dante, (b) reaction times are longer for incorrect responses than for correct responses, and (c) reaction times POSITIVELY correlate with metric plausibility.

 I believe the paper makes a significant, original contribution to the literature and can pass peer review with very minor clarifications and additions.

 GENERAL REMARKS:

1. While the research is framed around the question of meter's role in memory retention, it is worth emphasizing that the experiment does not compare meter with non-meter. Rather, the comparison is between meter and "almost-meter". A passage that has been manipulated to make it less regular in one respect is still regular in its other properties. The authors could discuss whether this issue makes their claims stronger (because their approach isolates specific aspects of meter) or weaker (because it does not test entirely unmetrical passages).
2. If I understand the systems right, the requirements of rhyme and syllable count are absolute, i.e. a line deviating from the 11-syllable count and the ottava or terzina rhyme simply could occur in Dante or Ariosto. On the other hand, accent distribution, other than in the 10th syllable, is not strictly regulated, and various accent patterns may be more or less likely but not absolutely unmetrical. It is then interesting that violations of absolute requirements in the NPS conditions are not ranked worse than violations of soft requirements (NPA). Does this mean that the subjects of the experiments are not in fact proficient in the knowledge of the relevant metrical systems and were not in fact perceiving the intended metrical structure, especially with reference to syllable count? Does this make the results of the paper weaker?

SPECIFIC REMARKS:

1. Where in the lines were the muted non-words? In rhyming position? in the middle? Were the muted non-words the ones causing metrical irregularity in the manipulated passages?
2. More details should be provided on how accent was manipulated in the NPA condition, and how that manipulation relates to the actual preferences or requirements of Dante's and Ariosto's meters.
3. It is odd to refer to rhyme as a subcategory of meter. Normally "meter" refers to the distribution of prominences (accents, syllable weight) in a line. Perhaps "formal" is a better cover term for both meter and rhyme, and "formal plausibility" could be used instead of "metrical plausibility".

If any, are all the source data and materials underlying the results available?

Yes

Is the work original in terms of material and argument?

Yes

Is the work clearly and cogently presented?

Partly

Is the argument persuasive and supported by evidence?

Yes

Does the research article contribute to the cultural, historical, social understanding of the field?

Yes

Does it sufficiently engage with relevant methodologies and secondary literature on the topic?

Yes

Reviewer Expertise:

Phonology, historical linguistics

I confirm that I have read this submission and believe that I have an appropriate level of expertise to confirm that it is of an acceptable scientific standard.

# Response

**Published:** 2022-12-21

**Authors:** Alessandro Treves

**Affiliations:** SISSA, Italy

*GENERAL REMARKS:*

1. *While the research is framed around the question of meter's role in memory retention, it is worth emphasizing that the experiment does not compare meter with non-meter. Rather, the comparison is between meter and "almost-meter". A passage that has been manipulated to make it less regular in one respect is still regular in its other properties. The authors could discuss whether this issue makes their claims stronger (because their approach isolates specific aspects of meter) or weaker (because it does not test entirely unmetrical passages).*
  We thank the reviewer for bringing up this important point which, with the comments by the other reviewers, made us realize that we had not emphasized enough our efforts to make the manipulations as soft as possible, to keep the text “almost” metrically correct and try to stay within a presumed linear regime. This is now clearer in the main text, and particularly with the addition of the new Fig.8 (in the Extended Data), that shows, through a comparison with shuffled responses, that the effects of the manipulations on plausibility and memory score are essentially small, and hence allow us to focus on their correlation.
2. *If I understand the systems right, the requirements of rhyme and syllable count are absolute, i.e. a line deviating from the 11-syllable count and the ottava or terzina rhyme simply could occur in Dante or Ariosto. On the other hand, accent distribution, other than in the 10th syllable, is not strictly regulated, and various accent patterns may be more or less likely but not absolutely unmetrical. It is then interesting that violations of absolute requirements in the NPS conditions are not ranked worse than violations of soft requirements (NPA). Does this mean that the subjects of the experiments are not in fact proficient in the knowledge of the relevant metrical systems and were not in fact perceiving the intended metrical structure, especially with reference to syllable count? Does this make the results of the paper weaker?*
  Indeed, we regard this as an interesting and somewhat unexpected result of our study. Although syllable number and rhyme are absolute requirements only in theory (a bag of acceptable tricks is available to occasionally elude them) we did select passages where they apply strictly, and it is true that it is much simpler to write a sort code to check them out, rather than to assess, with a computer, the validity of an accent pattern. Because of that, we took extra care in quantifying, as presented in Fig. 3, the amount of variability in those patterns in our material. Still, the intended recipients of Dante’s and Ariosto’s poetry were not computer codes, but roughly speaking the ancestors, with probably less formal education, of our subject cohorts.

 
*SPECIFIC REMARKS: *

1. *Where in the lines were the muted non-words? In rhyming position? in the middle? Were the muted non-words the ones causing metrical irregularity in the manipulated passages?*
  Thank you for noting this point, which we had left unclear. We carefully avoided the rhyming positions and those causing the other metrical irregularities. We have now specified in the Study Phase section: Muted non-words were usually positioned in the third verse. and further below The alternatives to the correct non-word were chosen by maintaining the same number of syllables, and the same accent. Typically, stems and intermediate vowels or consonants changed. Again, target non-words were generally in the third verse, aiming not to overload working memory from the moment they listened to the silent word until the test time. In a few cases where this was not possible (e.g., because there were no appropriate non-words in the third verse) a non-word in the second verse was chosen, towards the end. Notably, for every passage we chose options which were consistent across all conditions, allowing a fair comparison in the results. 
2. *More details should be provided on how accent was manipulated in the NPA condition, and how that manipulation relates to the actual preferences or requirements of Dante's and Ariosto's meters.*
  We are aware that accent device was the trickiest one. However, it has a crucial role in the Italian poetic tradition. We addressed this issue at our best, by consulting an expert and by referring to an Italian database collecting several literary masterpieces, and their metrical annotation. This is described also in the text: This manipulation was particularly non-trivial, since accent patterns are not rigidly defined. However, to validate proper original accents, we consulted with an expert scholar for *Orlando Furioso*, whereas for *Divina Commedia* we referred to the “Archivio Metrico Italiano”, a database collecting masterpieces of Italian literature with their accents annotated ( www.maldura.unipd.it). On these bases, we altered the “original accents” by putting them in different positions within the verse, taking advantage in particular of non-words with no mandatory accent.
3. *It is odd to refer to rhyme as a subcategory of meter. Normally "meter" refers to the distribution of prominences (accents, syllable weight) in a line. Perhaps "formal" is a better cover term for both meter and rhyme, and "formal plausibility" could be used instead of "metrical plausibility".*
  We now better clarify in the text that we refer to “meter” (the word we would mostly use in everyday conversation) in a broader sense.
